# Glycans in autophagy, endocytosis and lysosomal functions

**DOI:** 10.1007/s10719-021-10007-x

**Published:** 2021-08-14

**Authors:** Fulvio Reggiori, Hans-Joachim Gabius, Massimo Aureli, Winfried Römer, Sandro Sonnino, Eeva-Liisa Eskelinen

**Affiliations:** 1 Department of Biomedical Sciences of Cells and Systems, University of Groningen, University Medical Center Groningen, Groningen, The Netherlands; 2 Faculty of Veterinary Medicine, Institute of Physiological Chemistry, Ludwig-Maximilians-University Munich, Munich, Germany; 3 Medical Biotechnology and Translational Medicine, University of Milan, Milano, Italy; 4 Faculty of Biology, University of Freiburg, Freiburg Institute for Advanced Studies, and Signalling Research Centres BIOSS and CIBSS, Freiburg, Germany; 5 Institute of Biomedicine, University of Turku, Turku, Finland

**Keywords:** Endolysosomal system, Glycoproteins, Glycolipids, Glycoconjugates, Lectins, Sugar code

## Abstract

Glycans have been shown to function as versatile molecular signals in cells. This prompted us to look at their roles in endocytosis, endolysosomal system and autophagy. We start by introducing the cell biological aspects of these pathways, the concept of the sugar code, and provide an overview on the role of glycans in the targeting of lysosomal proteins and in lysosomal functions. Moreover, we review evidence on the regulation of endocytosis and autophagy by glycans. Finally, we discuss the emerging concept that cytosolic exposure of luminal glycans, and their detection by endogenous lectins, provides a mechanism for the surveillance of the integrity of the endolysosomal compartments, and serves their eventual repair or disposal.

## Introduction

### The routes to lysosomes: endocytosis and autophagy

Lysosomes are intracellular, acidic organelles surrounded by a single membrane, in which the catabolic processing of macromolecules takes place. They were described for the first time by Christian de Duve in the 1950s [1, 2]. Lysosomes are ubiquitously distributed in all eukaryotic cells and appear, by electron microscopy, as dense cytosolic bodies of heterogeneous size and morphology, mainly localized in the perinuclear region of the cell. The degradative roles of lysosomes strongly rely on distinct transport routes that allow cargo delivery into their interior from both intracellular and extracellular locations. Some of these trafficking pathways are also involved in the transport of resident enzymes and other (glyco)proteins that are essential for lysosomal functions.

**Fig. 1:**
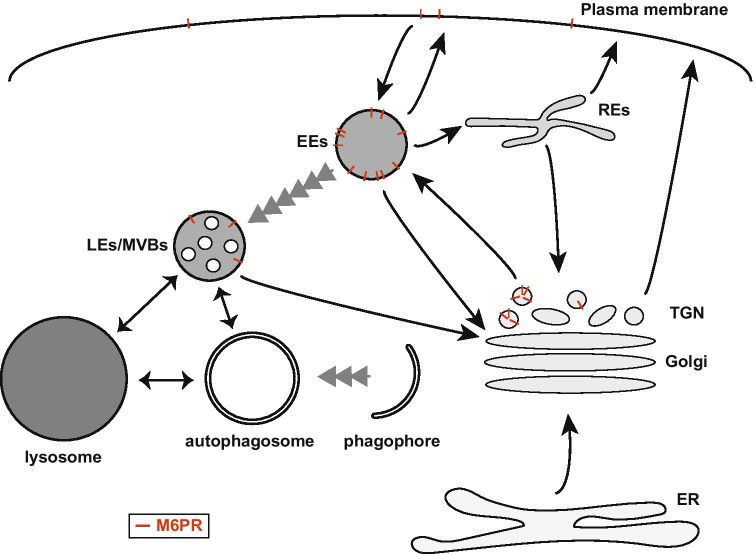
Simplified overview of the secretory and endolysosomal systems, and the principal glycoprotein transport routes. The synthesis of signal-peptide marked soluble and transmembrane (glyco)proteins on the rough endoplasmic reticulum (ER) leads to their translocation into the ER lumen or membrane, respectively. While some of the nascent proteins possess retention signals to let them remain localized in this compartment, others are transported to their final destination through sorting mechanisms that rely on the presence and/or absence of targeting signals that are encoded in peptide motifs and glycans. Integral plasma membrane and secreted (glyco)proteins reach the cell surface by vesicular traffic, passing through the Golgi. Endocytosed (imported) (glyco)proteins are delivered to the early endosomes (EEs), which are also the principal arrival point for newly synthesized (glyco)proteins destined to the endolysosomal system, sorted out from the secretory pathway at the trans-Golgi network (TGN). EEs are an important hub for (glyco)protein sorting and distribution. Lysosomal protein-sorting receptors can be recycled from EEs back to the Golgi for re-use, while plasma membrane proteins can be trafficked to the plasma membrane directly or via recycling endosomes (REs). Lysosomal soluble and transmembrane (glyco)proteins, however, remain associated with the EEs, and reach lysosomes through the maturation of EEs into late endosomes/multivesicular bodies (LEs/MVBs), which are characterized by the formation of intraluminal vesicles. LEs/MVBs finally fuse with lysosomes to deliver their membrane-associated and luminal cargos to this organelle. Autophagosomes arise through the generation and expansion of a phagophore, and when complete, they fuse either first with LEs and then lysosomes or directly with lysosomes. Arrows indicate the direction of main transport routes. Double-head arrows highlight fusion events between organelles. Serial arrowheads designate maturation processes. The Man-6-phosphate receptors (M6PRs, red bars) are highlighted in the compartments through which they are trafficking. M6PRs are mostly present in the TGN and EEs, but they can also be detected at the plasma membranes and LEs/MVBs

After synthesis in the endoplasmic reticulum (ER), lysosomal (glyco)proteins traverse the Golgi complex to enter the trans Golgi network (TGN), from where the majority of soluble lysosomal enzymes and lysosomal membrane proteins (LMPs) are transported to the endolysosomal system (Fig. 1). Most of the soluble lysosomal enzymes interact with sorting receptors to enter clathrin-coated vesicles traveling directly from the TGN to the early endosomes (EEs). This sorting step requires the heterotetrameric adaptor protein complex 1 (AP-1) and the Golgi-localized, $\gamma$-ear-containing and ARF-binding (GGA) proteins, which recognize specific motifs in the cytosolic tail of the sorting receptors and promote the subsequent formation of clathrin-coated vesicles [3–5]. The clathrin–coated vesicles can also sort LMPs from TGN to EEs [6]. In addition, a clathrin-independent transport route that delivers LMPs, but not sorting receptors, directly from the TGN to LEs has been discovered (Fig. 1) [7, 8]. Newly synthesized lysosomal enzymes and LMPs that escape sorting in the TGN enter the default secretion pathway to the plasma membrane and are targeted to lysosomes via endocytosis (Fig. 1). Low levels of the sorting receptors at the plasma membrane thereby mediate the endocytosis of the secreted hydrolases [9]. Although these transport routes are mainly biosynthetic, non-functional proteins can be recognized in the Golgi and ubiquitinated to be diverted into the endosomal system for lysosomal degradation [10]. Moreover, there are also transport pathways that directly transport proteins from the ER to LEs/lysosomes for turnover, to eliminate regulators of the ER associated degradation [11] or proteasome-resistant polymers of $\alpha$1-antitrypsin Z polymers [12].

Endocytosis is the major cellular pathway for internalization of extracellular material and begins with the formation of an endocytic vesicle (Fig. 1). There are several distinct subtypes of endocytic vesicles that bud from the plasma membrane and mediate entry into the cell. These differ by cargo and machinery, and include dynamin- and/or clathrin-dependent and independent pathways [6]. The commonly accepted model is that endocytic vesicles subsequently fuse with each other or directly with pre-existing EEs (Fig. 1). Ras-associated binding (RAB) GTPases are small monomeric G proteins that can switch between an active (GTP-loaded) and an inactive (GDP-loaded) state, which provide the means to spatially and temporally control intracellular traffic and signaling, but also organelle identity. EE-localized RAB5 is considered the *master regulator* of the endocytic pathway and exerts its action by attracting a variety of effector proteins [13, 14]. While soluble endocytosed cargos localize in the EE lumen, internalized integral membrane cargo (glyco)proteins destined for degradation in lysosomes, such as the epidermal growth factor and growth hormone receptors, are retained in the EEs by their incorporation into intra-luminal vesicles that form through the budding of the endosomal limiting membrane. Intra-luminal vesicle formation starts in the EEs and continues in LEs (Fig. 1) [14]. The process of cargo selection and intra-luminal vesicle formation involves the ubiquitin-dependent endosomal sorting complexes required for transport (ESCRT) complexes, but also other mechanisms have been described [6]. EEs mature into LEs through a mechanism that involves multiple rounds of membrane fusion and fission during which the protein and lipid composition of the EEs change as they acquire more intra-luminal vesicles (Fig. 1). Since LEs contain numerous intra-luminal vesicles, they are often also termed multivesicular bodies (MVBs). During the EE to LE maturation, RAB5 is replaced by RAB7 and the phosphoinositide phosphatidylinositol-3-phosphate (PI3P) is converted into phosphatidylinositol-3,5-biphosphate through phosphorylation [15, 16]. Moreover, soluble lysosomal enzyme-sorting receptors and other transmembrane proteins like soluble *N*-ethylmaleimide-sensitive factor attachment protein receptors (SNAREs) can be returned to the plasma membrane or transported to either the recycling endosomes (REs) or the TGN for reuse. LEs/MVBs can fuse with other LEs/MVBs or with lysosomes (Fig. 1) [14]. REs are generally identified by the presence of RAB4 and RAB11, and consist of a network of branched tubules with multiple (clathrin-coated) buds, which form exits from which cargo proteins can travel to distinct cellular destinations (Fig. 1) [14]. While most pathways emerging from REs divert from the degradative track and deliver proteins either to the plasma membrane or the TGN, RE can also be an intermediate station *en route* to the lysosomes since transmembrane proteins can be transported to the LEs/lysosomes in an adaptor protein AP-3-dependent manner (Fig. 1) [17].

Autophagic pathways deliver cytoplasmic material and organelles to lysosomes for degradation and recycling; the degradation products are exported from the lysosomes back to cytoplasm to be used as building blocks in biosynthetic processes and/or for energy production. Autophagy is an evolutionarily conserved catabolic process that supports the survival of eukaryotic cells during starvation but also other stresses. There are three distinct routes from the cytoplasm to lysosomes, which are called *macroautophagy*, *microautophagy* and *chaperone-mediated autophagy*. In macroautophagy, often simply referred to as autophagy, the cytoplasmic cargo is first sequestered into double-membrane vesicles called *autophagosomes* and then transported to lysosomes for degradation by fusion of the autophagosomal outer limiting membrane with a lysosome (Fig. 1) [18]. In microautophagy, the lysosomal or endosomal limiting membrane internalizes small portions of cytoplasm by invagination and subsequent pinch off, a process that leads to the formation of internal vesicles. These are eventually degraded with the cargo. In chaperone-mediated autophagy, a subclass of cytoplasmic proteins that have a KFERQ-like amino acid motif are first recognized by a cytosolic chaperone heat shock cognate 71 kDa protein (HSC70). The chaperone-cargo complex then interacts with lysosomal-associated membrane protein 2A (LAMP2A) on the lysosomal membrane, before the cargo protein is transported, in an unfolded state, through the lysosomal membrane [19]. Autophagy has diverse roles in cellular housekeeping including removal of damaged organelles, intracellular pathogens and harmful aggregates [20, 21]. Studies in several model organisms show that functional autophagy extends lifespan, which is likely connected to the prevention of metabolic stress and to the clearance of harmful proteins and worn-out organelles [22, 23]. Further, autophagy or its dysfunction have been implicated in many diseases including cancer, muscle and heart diseases, neurodegenerative and metabolic disorders, inflammation, and susceptibility to infections [24, 25]. Notably, autophagy may have both positive and negative effects on cancer progression, depending on the context [26–28].

**Fig. 2:**
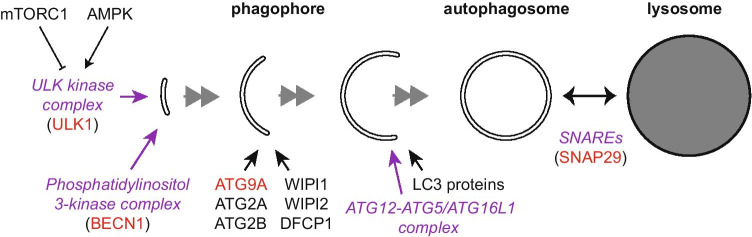
Locations of key autophagy protein functions during autophagosome biogenesis. Purple italic font indicates protein complexes or functional clusters; proteins in parenthesis are parts of the complexes or clusters. Red font indicates proteins that are *N*-glycosylated (autophagy-related 9A, ATG9A), or known to be regulated by Glc*N*Acylation (unc-51 like autophagy activating kinase, ULK1; beclin 1, BECN1 and synaptosome associated protein 29, SNAP1 29). mTORC1 mammalian target of rapamycin complex 1; AMPK AMP-activated kinase; ATG2, 12, 5, 16L1 autophagy-related 2, 12, 5, 16L1; DFCP1 double FYVE-containing protein 1; LC3 microtubule-associated light chain 3

Autophagosomes are formed by *phagophores*, flat membrane cisterns that elongate and curve into vesicles that are limited by two lipid bilayers (Fig. 2). Phagophore assembly is initiated when the unc-51 like autophagy activating kinase (ULK) complex, containing ULK1 or ULK2, autophagy-related 13 (ATG13), ATG101 and focal adhesion kinase family interacting protein of 200 kD (FIP200), is recruited nearby the ER [29–31]. ULK1 is activated during amino acid starvation, when the amino acid and energy sensors mammalian target of rapamycin complex 1 (mTORC1) and AMP-activated kinase (AMPK) are inactivated and activated, respectively [32]. The ULK kinase complex in turn activates the phosphatidylinositol 3-kinase complex I through direct phosphorylation. This complex contains beclin 1 (BECN1), phosphatidylinositol 3-kinase catalytic subunit type 3 (PIK3C3/VPS34, a PI3P-kinase), phosphoinositide-3-kinase regulatory subunit 4 (PIK3R4/VPS15), nuclear receptor binding factor 2 (NRBF2) and ATG14L, which positions the complex adjacent to the ER. Localized synthesis of PI3P on the phagophore recruits PI3P-binding proteins such as double FYVE-containing protein 1 (DFCP1) and WD repeat domain, phosphoinositide interacting 1 (WIPI1) or WIPI2 that collectively lead to the formation of ER subdomains called *omegasomes* [33]. Autophagosome biogenesis occurs in, or next to, omegasomes. Via interaction with ATG16L1, WIPI2 recruits the ATG12—ATG5 conjugate, which forms large oligomers by first binding to ATG16L1 and then multimerizing [34]. The ATG12—ATG5-ATG16L1 complex determines the site where the members of the microtubule-associated light chain 3 (LC3) protein family are conjugated to phosphatidylethanolamine (PE). The generated LC3-II is needed for the recruitment of LC3 proteins to the phagophore for different functions, including the closure of the phagophore into an autophagosome. The conjugation reactions of ATG12 to ATG5 and LC3 proteins to PE are assisted by ATG7 and ATG10, and ATG7 and ATG3, respectively. Upon autophagosome completion, the LC3-II pool on the outer limiting membrane dissociates, while the LC3-II in the inner limiting membrane is delivered to the lysosome together with the cargo.

### Protein and lipid glycosylation

The phylogenesis route to multicellular organisms has been paved by an increasing degree of sophistication of intracellular compartmentalization and vesicular transport. As outlined above, newly synthesized (glyco)proteins can reach intracellular compartments like lysosomes or the plasma membrane from the ER. The glycoproteins present in intracellular compartments are luminal or integrated into membranes, with their glycans mostly facing the lumen. Molecular signals for damage and ‘postal codes’ for reliable segregation ensure the intracellular system’s proper functioning. Thus, efficient surveillance mechanisms are needed to spot the glycans as indicators of intracellular membrane damage and subsequently, to initiate repair or removal by degradation. Here glycans with their exceptional talents come into play.

In contrast to nucleic acids and proteins, the linear sequence of a glycan, for example the disaccharide lactose composed of galactose (Gal)-glucose (Glc), is insufficient to define its structure. In addition, glycans are attached to carriers in a versatile manner to generate glycoproteins and glycolipids, underscoring the assumption of broad-scale versatility [35–37]. More information is required to define the structure of a glycan, i.e. on the position of the connecting glycosidic bond on both sugar units, and the nature of anomery (α or β). Adding this information turns Gal-Glc into Galβ1,4Glc to precisely define lactose, since this sugar is just one of the Gal-Glc-based disaccharides. These two structural parameters let glycans give an unsurpassed diversity among biomolecules, which upholds the use of the word *complex carbohydrates* [38], and explains why they are much *more complex, variegated and difficult to study than proteins or nucleic acids* [39]. In terms of encoding biological information, however, the possibility to generate a large panel of molecular signals by a small set of letters is ideal to generate a glycan-based mode of intra- and intercellular communication [40, 41]. Thus, sugars can be viewed as the third alphabet of life, following nucleotides and amino acids as the first and second letter sets.

In the context of intracellular trafficking, the sugar code is expected to be ubiquitous in eukaryotes, to require a highly organized and dynamically regulatable enzymatic machinery and to make the tagged proteins available for packaging into vesicles. All these expectations are entirely fulfilled by the co- and posttranslational route of *N-*glycosylation. It begins at the main entry site of the cellular membrane system, the ER, and proceeds in the Golgi apparatus, before the acceptor (glyco)proteins arrive at their final destination, which can be one of these organelles but also the plasma membrane or one of the compartments of the endolysosomal system [42–49]. The *N-*glycans of glycoproteins (and also other types of protein glycosylation) are thus a highly versatile biochemical modification to store biological information. In order to *translate* the information of glycan-encoded signals into a distinct bioactivity, this has to be *red* by sugar-specific receptors called lectins, a term derived from Latin *legere* [50] (for overviews of lectin history, classification and occurrence, please see [51, 52]). Since more than a dozen protein folds have developed the ability to bind glycans, and the encoding genes were platforms for duplications and intrafamily diversifications [53, 54], the versatility of glycan structure is matched by a large diversity in lectins with different glycan binding specificities. In fundamental terms, oligosaccharides can readily convey biorelevant information through their interplay with lectins.

Mono- and/or oligosaccharides are attached to proteins mostly through linkage to Ser/Thr or Asn residues, to lead to the so-called *O*- and *N-*glycosylations, respectively. Their biosynthesis, however, differs. While the constituents of *O*-glycans are added step-wise, *N-*glycan synthesis starts by a block-wise transfer. In particular, *N-*glycans are preassembled as a 14mer oligosaccharide (Glc_3_Man_9_Glc*N*Ac_2_) on a dolichol carrier initially on the cytoplasmic surface of the ER membrane, which subsequently translocates into the luminal side of the ER [55]. Once on the protein, this *N-*glycan is trimmed down step-wise to a heptasaccharide (Man_5_Glc*N*Ac_2_) in the ER, and finally re-elongated into the diversity of mature *N-*glycans by a series of glycosylation steps taking place in the Golgi cisternae [55]. Nine different types of *O*-glycosylation are found on proteins trafficking through the secretory pathway in vertebrates, and most *O*-glycans are further elongated/branched in the Golgi by sequential addition of monosaccharides by distinct enzymes [56]. *O*-Linked protein glycosylation can also take place on nuclear and cytosolic proteins. This is a dynamic event in which a monosaccharide such as fucose (Fuc) or *N*-acetylglucosamine (Glc*N*Ac), which does not get branched, is added in a process called Glc*N*Acylation, and removed from protein in a regulated manner to modulate its function [57–60].

Saccharides can also be linked to sphingolipids, yielding the broad class of glycosphingolipids (GSLs). The process starts with addition of a monosaccharide to a ceramide moiety, and such a small headgroup is already bioactive [61–63]. The sequential addition of monosaccharides leads to the formation of GSLs [64]. This class of lipids resides principally at the plasma membrane and reaches this location through the secretory pathway. Like in the case of the *N-*glycans, GSL glycans are generated and extended in the lumen of the ER and Golgi, leading to a topological distribution of GSL on the extracellular side of the plasma membrane [64]. Another lipid class that contains a glycan core are the glycosylphosphatidylinositol (GPI)-anchors [65, 66]. Those are assembled on phosphatidylinositol moieties with the sequential addition of one Glc*N*Ac and four mannoses (Man), but also ethanolamine groups, in the ER lumen [65]. GPI-anchors are conjugated to the C-terminus of a subset of newly synthesized (glyco)proteins translocated in the ER, which initially have a transient hydrophobic C-terminal peptide that is later removed. GPI-anchoring allows soluble proteins both to be associated to membranes and to reach the plasma membrane through the secretory route. GPI-anchored (glyco)proteins face the extracellular milieu at the plasma membrane [65].

## Glycans and lysosomes

### Lysosomal proteins are glycosylated

Lysosomes, as noted above, are delimited by a single membrane with a thickness of 7–10 nm, which is characterized by a continuous luminal glyco-layer generated by the presence of highly glycosylated LMPs [67]. It was originally suggested that the coating by sugars could have a protective role against the catabolic action of lysosomal hydrolases, but it has also become clear that the saccharide moieties are fundamental for the proper function of the LMPs in lysosomal motility, transport of catabolites across the membrane, and fusion processes occurring between lysosomes and others intracellular organelles and plasma membrane [68, 69]. Among LMPs, the most abundant are LAMP1 and LAMP2, which are characterized by more than 25 glycosylation sites and considered as lysosomal marker proteins. Interestingly, the glycan panel of these proteins contains *O*-glycan chains with *N*-acetyllactosamine repeats and sialyl Le^x^ (sLe^x^) termini, which are known to interact with cell adhesion molecules such as selectins [70]. In addition, *N*- and *O*-glycans are modified in almost equal efficiency to express sLe^x^ structures, suggesting that both *O*-glycans and *N-*glycans contribute to the expression of sialyl Le^x^ structures in LAMP proteins [71]. LAMP1 and LAMP2 represent 50% of all membrane proteins of the lysosomal membrane. Another important protein associated with the lysosomal membrane is the multisubunit vacuolar ATPase proton pump, which is responsible for the acidification of the lysosomal lumen [72]. Acidic intra-lysosomal pH is essential for the activation and function of the lysosomal enzymes. Lysosomes contain about 60 different acidic hydrolases involved in the degradation of specific substrates. They are mainly soluble except for those involved in the lipid catabolism, which are principally associated with the lysosomal membrane. Lysosomal enzymes are members of several classes of proteins such as proteases, glycosidases, phosphatases, sulphatases, lipases, and nucleases. This variety reflects the capability of the lysosomes to degrade multiple types of macromolecules, including proteins, membranes, nucleic acids and all kinds of glycoconjugates [73].

### Lysosome biogenesis is regulated by TFEB

Recent evidence supports the role of the transcription factor EB (TFEB) as master regulator in the transcriptional control of lysosomal hydrolases [74]. TFEB belongs to the microphthalmia family of basic/helix-loop-helix/ leucine zipper transcription factors (MiT family) [75, 76]. In particular, TFEB directly binds to a common 10-base E-box-like palindromic sequence of DNA called coordinated lysosomal expression and regulation (CLEAR) motif, which is found in one or more copies in the promoter of several genes encoding lysosomal proteins as well as ATG proteins (namely the CLEAR network) [74]. The activity of TFEB is strictly dependent on its phosphorylation at Ser211, which is the docking site for the chaperone 14-3-3, responsible for the sequestration of TFEB in the cytosol, preventing its nuclear translocation [77]. The main kinases responsible for TFEB phosphorylation are mTORC1 [78] and the extracellular signal-regulated kinase 2 (ERK2/MAPK1) [79]. In the presence of nutrients, small recombination activating (RAG) GTPases are active and recruit mTORC1 onto the lysosomal membrane, promoting its activation [80]. Upon nutrient deprivation or lysosomal stress, mTORC1 is released from the lysosomal membrane and becomes inactive. These events also induce Ca^2+^ release from lysosomes through the Ca^2+^ channel mucolipin 1 (MCOLN1) [81]. The increase in cytosolic Ca^2+^ concentration activates the phosphatase calcineurin, which dephosphorylates TFEB and promotes its nuclear translocation [81].

### Targeting of lysosomal proteins

After transcription, lysosomal enzymes are co-translationally translocated into the ER membrane or ER lumen, and subsequently processed in the Golgi apparatus before being sorted to reach lysosomes. Most of the soluble lysosomal enzymes acquire high-Man- or hybrid-type *N-*glycans that are characterized by the presence of Man-6-phosphate residues at distinct positions of the oligosaccharide branches [82]. The Man-6-phosphate tags are recognized by two types of specific Man-6-phosphate receptors (M6PRs) in the TGN (Fig. 1) [83]. M6PR-enzyme complexes are transported to EEs through clathrin-coated vesicles before reaching lysosomes. In the EEs, the increased acidity induces the release of the enzymes from M6PRs, which are then recycled back to the Golgi apparatus (Fig. 1). Interestingly, a different transport mechanism mediated by the lysosomal integral membrane protein 2 (LIMP2/SCARB2) has been identified for the lysosomal enzyme β-glucocerebrosidase, which is responsible for the hydrolysis of the simplest GSL at its glycosidic bond, i.e. glucosylceramide, in Glc and ceramide, a deficiency being the cause for the lysosomal storage disorder Gaucher’s disease [84]. Other LMPs, such as LAMP1 and LAMP2 are also not modified with Man-6-phosphate; their sorting depends on specific signal sequences that are mainly located in their cytosolic tails and/or transmembrane domains [5, 85, 86].

### Lysosomes have numerous functions in cells

The substrates to be catabolized can reach the lysosomes through two main pathways: endocytosis and autophagy. Although for a long time lysosomes have been considered the final destination for vesicular degradative pathways, it is now clear that they are also crucial regulators of cell homeostasis [87]. Lysosomes are involved in the repair of the plasma membrane through a mechanism involving Ca^2+^-mediated fusion [88, 89]. Another important process is lysosomal exocytosis that induces the release of the soluble lysosomal enzymes in the extracellular milieu, while enzymes anchored into the lysosomal membrane become components of the plasma membrane. In particular, the enzymes involved in the catabolism of sphingolipids act directly at the cell surface and fine tune specific pattern of these lipids in the plasma membrane [90, 91]. Lysosomes are also considered Ca^2+^ storage organelles; the concentration of this cation in their lumen is similar to that found associated with the Ca^2+^ storage organelles belonging to the ER [92]. Ca^2+^ is important to regulate different cellular processes, including trafficking, recycling and fusion.

Lysosomes also play a crucial role in cholesterol homeostasis. Cholesterol is an essential structural component of cellular membranes, and the majority of this lipid, i.e., 80% of its total cellular amount, is found in the plasma membrane where it constitutes about 40% of the total lipids [92]. Cholesterol is *de novo* synthesized in the ER, although an important amount can also derive from low-density lipoproteins (LDLs) via receptor-mediated endocytosis. In lysosomes, the action of acid lipases liberates non-esterified cholesterol from LDLs [93]. Cholesterol is then transported from lysosomes to other cellular compartments, such as Golgi apparatus, plasma membrane and ER, via two specific cholesterol-binding proteins, Niemann-Pick C1 (NPC1) and NPC2 [94].

Another interesting process is lysosomal-mediated cell death that occurs upon lysosomal membrane permeabilization followed by the release of hydrolytic enzymes into the cytosol [95]. In particular, the enzymes active at neutral pH, such as cathepsins B, D and L, are able to activate apoptotic effectors like mitochondrial proteins and/or cytosolic caspases. The features of lysosomal-mediated cell death, which can be necrotic, apoptotic or apoptosis-like, depend on the extent of the leakage and the cellular conditions.

Altogether, lysosomes are involved in numerous cellular processes and alterations in their functions have severe consequences on cellular homeostasis, which in turn lead to the onset of severe pathologies including hereditary lysosomal storage disorders.

## Glycans and endocytosis

### The different types of endocytosis

It was Christian De Duve in 1963, who first employed the term ‘endocytosis’ to describe the cellular uptake processes of macromolecules or fluids from the extracellular space into the cytoplasm [96]. Endocytic vesicles, which are generated from plasma membrane invaginations by budding, transport the ingested material to different intracellular compartments: LEs and lysosomes for degradation, REs for recycling back to the plasma membrane, or many other destinations in the cell such as the Golgi apparatus. At first glance, the size of the initial membrane invagination enables a classification into different endocytic pathways. The internalization of either large particles (such as bacteria, cell debris, or even intact cells) or large volumes of the extracellular bulk phase (relative to the cell volume) are tightly regulated processes called *phagocytosis* and *macropinocytosis*, respectively (reviewed in [97–99]). Smaller cargo can be internalized by a plethora of microscale endocytic pathways, which can operate concurrently at the plasma membrane [100]. Amongst them, clathrin-mediated endocytosis (CME) is the most studied and probably the best understood entry route into the cell (reviewed in [101–103]). In addition to CME, cells use a broad variety of alternative, heterogeneous entry routes, roughly classified as clathrin-independent endocytosis (CIE) pathways (reviewed in [104–106]). In contrast to CME, in which each cargo receptor has cytoplasmic sorting motifs leading to the assembly of a well-defined endocytic machinery that generates the clathrin coat, CIE deals with the internalization of various molecules, ranging from glycosphingolipids and GPI-anchored proteins to transmembrane proteins that all lack cytoplasmic domains and consequently also a cytoplasmic sorting motif. Even if each of the CIE pathways has several unique components, many of them share common features, such as association to lipid rafts, implication of cholesterol, actin polymerization and possibly glycosylation.

Many decades of research have shown that endocytosis extends far beyond the mere uptake of material from the extracellular space or the cell surface; it rather interconnects and regulates a multitude of cellular activities, including nutrient uptake, cell signaling, cell migration, cell polarity, antigen presentation and mitosis [107].

### The role of protein glycosylation in receptor-mediated endocytosis

The importance of post-translational modifications, such as phosphorylation or ubiquitylation, for receptor-mediated endocytosis is well-recognized (reviewed in [108, 109]). The pioneering work of Ashwell’s laboratory led to the purification of the first endocytic glycan receptor of vertebrates, called the hepatic asialoglycoprotein receptor, and also started the novel research area of mammalian glycan receptors [110–112]. Currently, the number of publications reporting that glycosylation influences the association of receptors with highly specific plasma membrane domains is steadily increasing. A few of these publications are highlighted in detail below.

Kohno and co-workers analyzed the glycosylation pattern of the sphingosine-1-phosphate (Sph-1-P) receptor endothelial differentiation gene-1 product (EDG1) and the influence of glycosylation on its internalization. Former studies had shown that in response to Sph-1-P binding, EDG1 triggers diverse signaling pathways through downstream signaling molecules, such as Ca^2+^ and mitogen-activated protein (MAP) kinases, by activating heterotrimeric G-proteins. EDG1 is glycosylated at its extracellular domain and one glycosylation site has been identified at Asn30. Although there was no difference in the ligand binding ability and ligand-induced MAP kinase activation between the wild-type and the non-glycosylated mutant Asn30Asp-EDG1, Asn30Asp-EDG1 was much less responsive to ligand-induced internalization. Moreover, in contrast to the wild-type receptor, Asn30Asp-EDG1 was no more associated with the CIE organelles called caveolae, but dispersed broadly in membrane fractions separated by sucrose density gradient centrifugation, suggesting that microdomain localization and internalization of *N-*glycosylated EDG1 might be connected [113].

Min and colleagues used the G protein-coupled dopamine D2 and D3 receptors, which are glycosylated at their extracellular domains, as experimental model to study the impact of glycosylation on receptor functions including cell surface expression, signaling, and internalization through specific plasma membrane domains. The results showed that the glycosylation on the N-terminus mediates the internalization of D2 and D3 receptors through caveolae and clathrin-coated pits, respectively, by regulating receptor interactions with caveolin-1 and clathrin [114].

Finally, Altschuler and co-workers observed that CME of the type I transmembrane protein mucin 1 (MUC1) is influenced by its glycosylation state. In particular, by comparing the internalization of various glycosylated forms of MUC1 in wild-type and glycosylation-deficient cells, they showed that the initial rate of endocytosis of the under-glycosylated MUC1 was stimulated twofold in comparison with normally glycosylated MUC1, while trafficking to lysosomes remained unaffected [115].

Altogether, these examples highlight that glycosylation of specific plasma membrane proteins plays an important regulatory role in their dynamics during endocytosis.

### Lectin-glycolipid interactions induce membrane curvature leading to tubular membrane invaginations

Glycans can act as ligands/receptors for glycan-binding proteins called lectins. Lectins and glycans play crucial roles in multiple physiological and pathological processes. For instance, lectin-glycan interactions are involved in the regulation of cell-surface receptors [116], immune cell migration [117], recognition of pathogens [118], pathogen infection [119] and in many other areas of biology and medicine. Hereby, either membrane-anchored or soluble lectins are responsible of decoding glycan-containing information. Table 1 depicts examples of lectin-glycan interactions that are implicated in diverse endocytosis pathways. The following paragraphs focus on the interactions of lectins with GSLs, which can impose various effects on plasma membrane organization and dynamics (reviewed in [120, 121]). In addition to cellular studies, the use of synthetic membrane systems, such as giant unilamellar vesicles or supported lipid bilayers, has contributed substantially to the understanding of the mechanistic details underlying lectin-GSL interactions (reviewed in [122, 123]).

**Table 1:** Examples of lectin-glycan interactions in endocytosis. The lectin, the lectin-expressing cell type, and the corresponding glycan interaction partner(s) are indicated

Lectin	Cell type/organism	Glycan/receptor	References
*C-type lectins*			
Asialoglycoprotein receptor (ASGR)	Mainly hepatocytes	Desialylated glycoproteins that containterminal Gal or GalNAc residues on theirN-linked glycans, and also sialylatedglycoproteins with terminal Siaα2,6GalNAcand Siaα2,6Gal	Reviewed in [265, 266]
CD209/DC-SIGN	Human myeloid dendritic cells	Branched D-Man and L-Fuc motifscommon on pathogen surfaces; e.g.,mannan, Lex and Ley	Reviewed in [267]
Langerin (CD207)	Mainly Langerhans cells	Man-rich *N*-glycans on proteins;e.g., HIV-1 gp120 envelope protein	[268]
Man-binding lectin	Mainly hepatocytes	Man-rich *N*-glycans	[269]
(MBL2)			
*I-type lectins (they bind to non-reducing terminal sialic acids)*			
Sialic acid binding Ig like lectin 1 (SIGLEC1/CD169)	Macrophages	e.g., HIV-1 gp120 envelope protein	[270]
CD22	B cells	Sialoside ligands on the surface of both the same cell and adjacent cells	[271]
*Galectins (they bind to β-galactoside-containing glycans)*			
LGALS1	e.g. T cells	e.g., PTPRC/CD45	[272]
LGALS3	e.g. fibroblasts	e.g., CD44 or glycosphingolipids	[148]
*Plant lectins/toxins*			
Ricin (R-type lectin)	*Ricinus communis*	Terminal β-linked Gal- or Gal*N*Ac-containing glycans	Reviewed in [273]
Gs I-A4	*Griffonia* (*Bandeirea*) *simplicifolia*	Terminal α‐linked Gal*N*Ac residues, including the Tn antigen	[274]
*Bacterial lectins/toxins*			
StxB	*Shigella dysenteriae *and *Escherichia coli*	Mainly Gb3	[136]
Cholera toxin	*Vibrio cholerae*	Ganglioside GM1, LeX on proteins	[275]
LecA	*Pseudomonas aeruginosa*	e.g., Gb3	[142]
RSL	*Ralstonia solanacearum*	e.g.*, *human histo-blood group antigens	[132]
*Viral lectins*			
VP1	Simian virus 40	Ganglioside GM1	[140]
VP1	Norovirus GII.4	ABH histo-blood group glycans on glycoproteins and GSLs	[141]

GSLs are mainly found in the extracellular leaflet of the plasma membrane. Their ceramide backbone is embedded in the lipophilic part of the membrane while the carbohydrate moieties are exposed to the extracellular space. The common view is that GSLs tend to associate with each other [124] and are enriched in lipid rafts, where they interact with cholesterol and sphingomyelin [125, 126]. Nevertheless, variations in the fatty acyl chain of the ceramide backbone, e.g. in length or saturation level, can affect the partitioning and orientation of GSLs in plasma membrane domains [127]. This, in turn, can influence the exposure of the carbohydrate chain rendering it partially inaccessible for binding to ligands [128, 129].

**Fig. 3:**
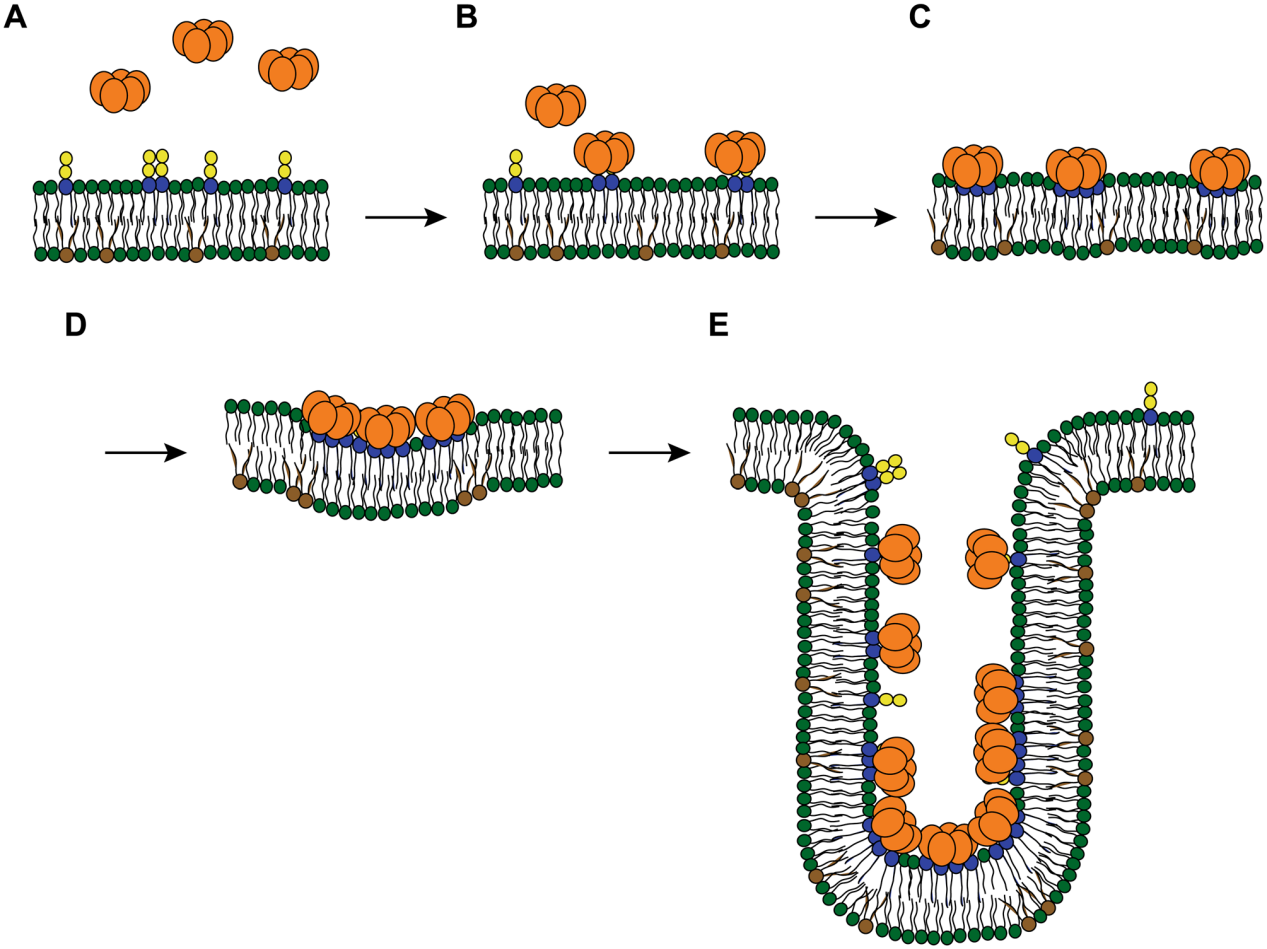
StxB induces tubular membrane invaginations. **A** The B-subunit of Shiga toxin (StxB, orange color) is recruited by the glycosphingolipid Gb3 (carbohydrates are colored in blue and yellow) onto the extracellular membrane leaflet of the plasma membrane. Initially, the pentameric StxB binds to individual Gb3 molecules **B** and subsequently, the StxB pentamers are able to cluster up to 15 Gb3 molecules, leading to increased affinity/avidity of interactions. This event leads to a small local negative membrane curvature **C**. Further Gb3 clustering and membrane reorganization processes **D** trigger the formation of narrow tubular membrane invaginations **E**. Lipids in green and brown color represent different phospholipids with saturated and unsaturated fatty acyl chains, respectively

Several lectins and toxins with a subunit acting as lectin, induce membrane curvature by binding to glycolipids in cells or synthetic membrane systems, leading to tubular membrane invaginations [130–135]. For instance, the homo-pentameric B-subunit of Shiga toxin (StxB), which is produced by *Shigella dysenteriae* and enterohaemorrhagic *Escherichia coli* strains, specifically recognizes the carbohydrate moieties of the GSL globotriaosylceramide (Gb3, also known as CD77 or the P^k^ blood group antigen). StxB can recruit up to 15 Gb3 molecules underneath its pentameric structure (reviewed in [136]). This multivalent binding leads to Gb3 clustering and local membrane reorganization, resulting in a small increment of negative membrane curvature [137, 138]. Subsequent membrane-mediated clustering of several StxB molecules creates a tubular plasma membrane invagination that paves the way of StxB into the cell [133, 134]. The molecular mechanism to generate membrane curvature and tubular invaginations is not yet fully understood, but it seems to require the multiplicity and specific architecture of GSL-binding sites (Fig. 3). In order to study the impact of these two crucial parameters on the formation of membrane invaginations, distinct carbohydrate-binding sites of diverse lectins have been modified by site-directed mutagenesis [132, 133, 135]. The StxB variant Trp34Ala, in which the Gb3-binding site III was inactivated by classical mutagenesis, only rarely induced membrane tubules in cells and not at all in giant unilamellar vesicles, even when used at high concentrations to ensure efficient binding [134].

A fully novel approach has been chosen for a more detailed mechanistic study on the induction of membrane invaginations. By linking the three subunits from *Ralstonia solanacearum* lectin (RSL), several monomeric so-called neolectins (neoRSLs) with different valences were designed, which are highly similar in structure to the wild-type lectin [139]. Compared to the unmodified trimeric and hexavalent lectin, already a divalent, monomeric variant was sufficient to induce tubular membrane invaginations in giant unilamellar vesicles by binding to glycolipids with a Fuc-containing glycan. However, a divalent variant with two adjacent carbohydrate-binding sites was able to form membrane invaginations, while divalent proteins with the binding sites further apart did not deform the membrane, even though their binding affinities were fairly similar [139]. Similar observations, i.e. membrane curvature and tubular membrane invaginations that can form without the assistance of an active cellular machinery, have also been made upon the binding of viral particles to GSLs [124, 140, 141]. For instance, the viral capsid of simian virus 40, which is composed of 72 VP1 pentamers, each one resembling the 3D-structure of the pentameric B-subunits of cholera or Shiga toxin, binds to the ganglioside GM1 promoting lipid compaction and membrane invaginations [140]. The bacterium *Pseudomonas aeruginosa* is an excellent example to highlight that also bacteria rely on lectin-induced GSL clustering as a strategy for host cell invasion. The interactions of the tetrameric *P. aeruginosa* lectin LecA with Gb3, its binding partner on host cell plasma membranes, trigger the cellular uptake of the bacterium into epithelial cells via the so-called lipid zipper mechanism [142].

In summary, even if the initial forces driving the membrane curvature might differ between several lectins and toxins with lectin properties, both viruses and bacteria exploit lectin-induced GSL clustering for cellular uptake and probably also for signaling [143, 144]. In addition to exogenous proteins such as bacterial toxins and viruses, tissue lectins are known to act in a similar manner.

### Galectins and glycans regulate endocytic processes

Galectins are a family of lectins that bind β-galactosides through a structural β-sandwich-fold domain ([145]; for a collection of reviews covering the broad range of the field, please see two special dedicated issues of *Trends in Glycosciences and Glycotechnology* [146, 147]). Galectins have numerous intracellular functions, and after non-classical secretion, they also have extracellular effects and are also involved in endocytosis [148–150]. Two rather controversial findings on the role of glycosylation and galectins in CIE have been reported. ‘Galectin lattice’, a large (cross-linked) interaction network brought together by galectins, was reported to sequester cargo molecules (e.g. several growth factor receptors) at the cell surface, thus inhibiting their cellular uptake [116, 151, 152]. Interestingly, a significant compaction of the LGALS1’s carbohydrate recognition domains (CRDs) upon ligand binding, which affects LGALS1 overall structure and increases the stability of the homodimers [153], is probably underlying the mechanism leading to the organization of rigid aggregates [154, 155]. On the contrary, galectin-3 (LGALS3/Gal-3), probably in its pentameric form, was reported to drive the internalization of CD44 in conjunction with GSLs, by bending the plasma membrane in a similar manner as reported for several bacterial toxins [148]. However, these apparently controversial findings, i.e., *inhibitory* versus *stimulatory* roles of a galectin, can be reconciled with each other, within the same cell line [150]. That is, the initial galectin-glycan interactions can stimulate CIE while an increasing number of galectin-glycan interactions leads to the assembly of a galectin lattice, which inhibits endocytosis. In light of recent findings, other functional oligomeric states of LGALS3 than pentamers, may exist via the dynamic self-association of its intrinsically disordered N-terminal domain [156]. In a very recent study, Farhadi and colleagues, using engineered LGALS3 constructs (from dimers to hexamers) based on α-helical coiled-coil scaffolds, confirmed that glycan-binding properties, clustering and extracellular signaling activities of LGALS3 depend on architecture and valency, extending previous work with engineered LGALS3 [157–159]. The authors of this study also highlighted that the relatively large integral membrane glycoprotein protein tyrosine phosphatase receptor type C (PTPRC/CD45) (in contrast to smaller glycoproteins such as CD7) regulates membrane glycan clustering and cell death signaling activities of synthetic LGALS3 oligomers [157]. Thus, parameters like the glycosylation pattern, galectin secretion, valency and quaternary structure have a major impact on galectin-glycan interactions, which in turn modulate the fate of diverse cargo proteins [150, 157].

In conclusion, the interactions between glycans and lectins appear to be a versatile strategy that cells employ not only to regulate endocytosis, but also to modulate intracellular signaling pathways, thus regulating cell migration, proliferation, apoptosis and other cellular processes.

## Glycans and autophagy

### ATG9A is an N-glycosylated transmembrane protein

ATG9A has been recognized as an essential protein for autophagosome formation for a long time (Fig. 2), but its molecular mechanisms were only recently recovered. ATG9A, unlike all the other core ATG proteins, is an integral membrane protein, which traffics between the TGN and endosomes [160]. It has four transmembrane segments and two helices that enter and exit the membrane on the cytosolic side, and both the C- and N-termini are cytosolic [161–163]. Vesicles containing ATG9A are required for phagophore assembly [164], and traffic to phagophore assembly sites from either the REs or TGN [165–168]. Recent results show that ATG9A vesicles function as seeds for phagophore biogenesis [169]. The cryo-electron microscopy structures of human ATG9A and fission yeast Atg9 were recently solved. The structure is a homotrimer that has a unique fold and an internal network of branched cavities [161–163]. Notably, the cavities form a path between the two leaflets of the lipid bilayer; ATG9A functions as a flippase that facilitates lipid translocation from the cytoplasmic to luminal leaflet of ATG9A-positive membranes, thereby enabling the elongation of nascent phagophores [162, 163]. Association of ATG9A with ATG2 proteins (ATG2A and ATG2B), is crucial for phagophore biogenesis [170]. ATG2A and ATG2B are rod-shaped lipid transfer proteins [171–174]. The current view is that ATG2 proteins transport lipids from the ER to the cytoplasmic leaflet of the growing phagophore membrane, and ATG9A then distributes the lipids to the luminal leaflet in order to facilitate phagophore elongation [175, 176]. ATG9A is glycosylated with a complex-type *N-*glycan at Asn99 between the first and second transmembrane segments; Asn99 is the only one of the potential *N*-glycosylation sites in ATG9A that is used [160, 177]. The presence of ATG9A is a possible explanation for the early observations of Yamamoto and colleagues, showing that the growing edges of phagophores have binding sites for concanavalin A, wheat germ agglutinin and *Ricinus communis* agglutinin 120, which bind Man, Glc*N*Ac and sialic acid, and terminal β-Gal residues, respectively [178]. However, the role of the ATG9A *N-*glycan in its trafficking and function at the phagophore remains totally unexplored.

### Glycolipids and Glc*N*Ac–modified proteins in autophagy

In addition to the transmembrane glycoprotein ATG9A, also glycolipids have roles in phagophore biogenesis. The ganglioside Gd3 colocalizes with LC3 proteins, suggesting it is present in autophagosomal membranes. Perturbations in Gd3 biosynthesis inhibit autophagosome biogenesis [179]. This glycolipid may contribute to the curvature and/or fluidity of the membrane, which are likely to modulate membrane dynamics during autophagosome biogenesis.

As mentioned above, attachment of Glc*N*Ac to the hydroxyl group of serine or threonine residues of proteins is a special type of glycosylation taking place in the cytoplasm [57, 180]. *O*-Glc*N*Ac transferase adds this sugar moiety while *O*-Glc*N*Acase cleaves it, and the modification responds to a variety of environmental conditions and acts as a stress signal in many intracellular processes including autophagy [180]. Like autophagy, *O*-Glc*N*Acylation is regulated by nutrient availability. The ULK1 kinase, which is central in autophagy induction, is modified by *O*-Glc*N*Ac at Thr754 during glucose starvation, and this modification is required for the activation of PIK3C3 and initiation of autophagosome formation [181] (Fig. 2). The central autophagy protein BECN1, which is part of the phosphatidylinositol 3-kinase complex complex I, was also shown to be modified by *O*-Glc*N*Ac, but in this case the modification was proposed to inhibit autophagy. Further, the levels of *O*-Glc*N*Acylated BECN1 were observed to be increased in the hearts of diabetic animals [182]. The authors of this study suggested that increased *O*-Glc*N*Acylation levels in diabetic heart may contribute to the abnormal autophagic response to Glc deprivation. The fusion of autophagosomes with endosomes and lysosomes is regulated by SNARE-proteins. One component of the SNAREs that regulate this fusion is synaptosome associated protein 29 (SNAP29), which is *O*-Glc*N*Acylated at four residues: Ser2, Ser61, Thr130 and Ser153. Preventing this modification in all four residues by point mutations enhances the formation of the SNAP29-containing SNARE complexes, thus increasing the fusion of autophagosomes with LEs and lysosomes [183]. Consistently, *O*-Glc*N*Acylation of SNAP29 is reduced during starvation, which promotes autophagosome maturation. Thus, *O*-Glc*N*Acylation of SNAP29 regulates autophagic flux according to the cellular nutrient status.

### Regulation of autophagy by extracellular glycoproteins and proteoglycans

Interactions of cell-surface receptors and extracellular glycoprotein ligands regulate intracellular pathways, including autophagy. Plasma membrane receptors bind soluble ligands as well as molecules attached to the extracellular matrix. Many soluble extracellular glycoprotein ligands such as decorin (DCN), thrombospondin 1 (THBS1), biglycan (BGN), endostatin (COL18A1), collagen VI and plasminogen kringle 5 activate autophagy in cancer cells, leading to inhibition of angiogenesis and tumor growth, while others such as laminin α2 chain (LAMA2) and lumican (LUM) are anti-autophagic [184–186]. Extracellular matrix-associated glycoprotein ligands mostly appear to repress autophagy. Attachment of cells to the basement membrane tends to restrict autophagic activity at a basal level, while cells detached from the basement membrane activate autophagy as a survival mechanism [187, 188]. Many plant lectins have shown potential as cancer treatments through autophagy modulation. As an example, concanavalin A treatment induces autophagy and cell death in U87 glioblastoma and HeLa epitheloid cervix carcinoma cells [189, 190]. The signals from extracellular glycoproteins and proteoglycans are thought to converge with common core autophagy machinery via their cell-surface receptors and autophagy-activating signalling pathways [186]. However, the detailed signalling routes that extracellular glycoproteins and plant lectins use to modulate autophagy have not been fully characterized (reviewed in [186, 191]).

## Glycans and protein quality control

*N-*Glycans as signal are an elegant solution for the quality control and transport of a glycoprotein. As noted above, all *N*-glycans initially have the Glc_3_Mac_9_Glc*N*Ac_2_ structure that is trimmed after quality control and then reshaped in the Golgi. Thus, *N*-glycans carry out three main tasks in the same way for all glycoproteins. First, they provide the assistance in correctly folding the proteins in the ER by being the docking site of the lectin chaperones calnexin (CANX) and calreticulin (CALR). Second, they label misfolded proteins for ER-associated degradation (ERAD) by a complex containing an F-box protein with lectin activity. Third, they are the molecular equivalent of a postal code for routing glycoproteins from the ER toward the Golgi and beyond by lectin cargo shuttles such as lectin Man binding 1 (LMAN1/ERGIC53), lectin Man binding 1 like (LMAN1L/ERGL), lectin Man binding 2 (LMAN2/VIP36) and lectin Man binding 2 like (LMAN2L/VIPL) [53, 192–199]. Within the lumen of the ER, Glc residues in *N*-glycans can be recognized by the lectin chaperones CALR and CANX, and this binding is not outcompeted by soluble Glc since Glc is absent at this location. As Gesner and Ginsburg noted in 1964, a recognition signal based on Glc would not work on the cell surface [200]. Cycles of deglucosylation/reglucosylation during chaperone-guided folding in the ER lumen ensure sufficient encounter of glycoproteins with the quality-control elements. Interestingly, a plethora of further functions in cancer biology is being unraveled for these ER chaperones [201].

Of particular relevance to illustrate variability, the CRDs of the lectins act in concert with other sections of the CRD, with other domains (in modular lectins) or with other subunits when lectins are part of a complex. This principle of modularity is known for the chaperones and their interaction with ER folding factors such as ERp57 (protein disulfide isomerase family member 3, PDIA3) via the P-domain [202]. Instead of different domains within a lectin working together, the CRD of LMAN1 pairs with a different protein, i.e. MCFD2, to interact with the cargo, possibly via its glycan and protein parts [203, 204]. Complex formation through bifunctional interaction also applies to F-box proteins to assemble the S-phase kinase-associated protein 1 (SKP1) – cullin-1 (CUL1) –F-box (SCF) E3 ubiquitin ligase complexes, a key effector in ERAD. The first F-box protein identified as lectin was the F-box protein recognizing sugar chain 1 (FBX02/FBS1), which displays specificity for the (Glc*N*Ac)_2_ stem in the *N-*glycan core and gets access to it in unfolded glycoproteins [198, 205, 206]. In addition to ubiquitination as a mode to label products of inadequate quality for ER-associated degradation, distinct SCF complexes can also trace glycoproteins that have already reached other compartments along the secretory pathway. For example, FBX027/FBS3 is the sensor part of the SCF complex that adds ubiquitin to glycoproteins that are exposed to the cytoplasm upon lysosomal membrane damage, to recruit the autophagy receptors such as sequestosome 1 (SQSTM1/p62) and to promote the autophagic turnover of the damaged organelle [198, 207]. The glycans of LAMP2 are the biorelevant binding partners of FBX027 in the context of lysosome damage [208]. Notably, the glycans of LAMP proteins are also recognized by another family of β-sandwich-type lectins, known as Gal-binding lectins or galectins (see below) [209, 210].

Homeostasis within the ER protein-folding factory and thus the flux of mature soluble and membrane (glyco)proteins is guaranteed by the unfolded protein response (UPR) [211–213]. Accumulation of misfolded and/or unfolded (glyco)proteins in the ER triggers a safeguard mechanism that is under the control of two UPR-specific transcription factors, X-box binding protein 1 (XBP1), and/or the activating transcription factor 6 (ATF6), as well as ATF4, another transcription factor. Those lead to the enhanced expression of the components of ER protein-folding machinery, including the ER-localized lectins, which leads to the upregulation of the chaperoning activity, and permits to restore the ER-based quality control. UPR also induces autophagy, in particular ER-phagy, to alleviate ER expansion induced by ER stress and also acts as an alternative disposal pathway for misfolded proteins [214]. Interestingly, the XBP1-triggered shifts in the *N*-glycan profile in cell line models such as HEK293 and HeLa, suggests the existence of a connection between the intracellular stress response and extracellular *N*-glycan functions [215, 216], but the physiological relevance of this phenomenon remains to be uncovered.

Overall, ER-resident lectins participate in the ER quality control and in routing glycoproteins to the Golgi and beyond, by associating with trimmed *N-*glycans and using more than one binding domain. The bifunctionality in binding is generated by either covalent association of the CRD and non-CRD modules within a single lectin, or by non-covalent assembly of a lectin with other proteins [217, 218]. Signaling damage of an endolysosomal compartment by recognizing cytoplasmic exposure of the *N-*glycans of LAMP2 represents a paradigm: galectin-9 (LGALS9/Gal-9) binds to the *N*-acetyllactosamine repeats in the *N-*glycan attached to Asn175 [210]. Of note, poly-*N*-acetyllactosamine stretches in *N*-glycans (preferably in β1,6-branches), mucin-type cores 2/4 *O*-glycans and keratan sulfate, are also high-affinity binders for (LGALS1/Gal-1) and galectin-3 (LGALS3/Gal-3) [219, 220]. That is, galectins are a candidate for *announcing that something is wrong*, after having detected mature *N-*glycans at an inappropriate place, i.e., reoriented or accessible in the cytoplasm [221–223]. This concept of a signal, a mature *N-*glycan becoming cytoplasmic, and its sensor, a cytoplasmic lectin endowed with bifunctionality, gave an incentive to look at galectin functions more closely.

## The roles of glycans in maintaining the integrity of the endolysosomal compartments

### Intracellular damage and galectins

The hypothesis for a role of glycans in the “*establishment of cell–cell contacts and possibly also as mediators of communication between the surface and the interior of the cell*” prompted researchers in Rehovot to apply an activity assay for detection of lectins, i.e. the haemagglutination reaction commonly used for testing plant extracts, on nervous tissue [224]. These experiments found a strong lectin activity in the electric organ of the electric eel *Electrophorus electricus*, as well as in rat, mouse and embryonic chicken tissues [224]. Since the presence of β-galactosides inhibited this lectin activity, affinity chromatography of tissue extracts on Gal-presenting resin yielded to the purification of the first galectin [224]. Further work along this line, and the biochemical analyses of the obtained proteins, revealed conserved amino acid sequences that are responsible for the key interactions with the cognate β-galactoside residues on glycans. This *sequence signature* is the common denominator of galectins and highlights the probable diversification of this type of β-sandwich proteins from an ancestral single gene into a protein family [225–229]. In addition to the sequence signature, the absence of a signal peptide sequence is another unique feature of the vertebrate galectins [227, 230, 231]. Thus, as opposed to the above-mentioned classes of β-sandwich-type lectins involved in quality control and cargo transport through the secretory pathway, galectins do not enter the ER and the endomembrane system. Their cytoplasmic presence qualifies them for the suggested assignment to control compartment integrity.

**Fig. 4:**
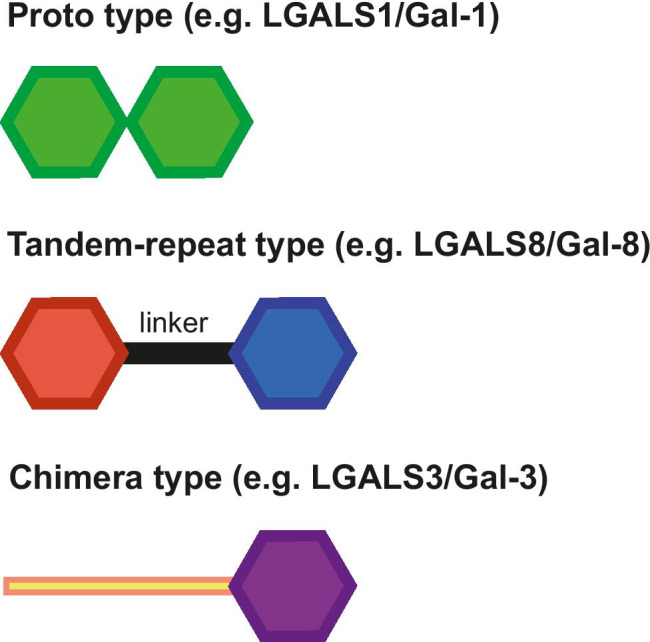
Schematic illustration of the three types of modular architecture of vertebrate galectins. The hexagon symbolizes the beta-sandwich-type carbohydrate recognition domain (CRD). Non-covalent association to a homodimer and conjugation of two different CRDs (as shown by the color coding) by a linker establish the proto type and tandem-repeat type, respectively. Extension of the canonical CRD by a tail comprising for example collagen-like repeats as in the case of galectin-3 (LGALS3), leads to the chimera type

To be intracellular sensors and initiators of a protective response, galectins must possess an in-built bifunctionality. Indeed, binding partners of galectins are on the one hand the glycoconjugates, glycoproteins, proteoglycans and glycolipids via their glycans, and on the other hand cytoplasmic and extracellular proteins (LGALS1 and LGALS3 interactors have recently been compiled [229]). The cooperative interplay between two different binding sites establishes the specific pairing between a specific glycan and particular protein so that a galectin can act like a molecular glue, bridging a glycan signal with an effector or modulator element. The prerequisite of serving as a molecular hinge is fulfilled by the three types of vertebrate galectins, i.e., non-covalently associated homodimers (i.e., proto types), linker-connected heterodimers (i.e., tandem-repeat types) and chimera composed by a CRD and an N-terminal tail with collagen-like repeats or other domains (i.e., chimera types) [159] (Fig. 4). For example, the chimera-type LGALS3 is engaged in phagocytic clearance of glycan-presenting apoptotic cells and cellular debris by simultaneously interacting with the glycans on the cargo and the MER proto-oncogene tyrosine kinase (MERTK) on the surface of phagocytic cells [232]. LGAL3 is also engeged in immune regulation via binding to cell surface glycans and the chemokine CXCL12 [233]. Homodimeric (proto-type) galectins such as LGALS1/Gal-1 alternatively mediate cross-linking by homo- and heterotypic interactions. In fact, the in-built “binding promiscuity” has been suggested to explain “the variety of adhesion phenomena by galectin-1” [234]. In contrast to tandem-repeat-type galectins such as galectin-8 (LGALS8/Gal-8) or LGALS9, which possess two different CRDs connected by a linker, the association of two CRDs in proto-type galectins is non-covalent (Fig. 4).

In summary, galectins have the properties for serving as guardians of intracellular organelle integrity, i.e., they are cytoplasmic and they specifically recognize their cognate glycans on a set of particular intraluminal glycoproteins. Involving more than a single galectin in surveillance of organelle integrity gives versatility to this system.

### The role of galectins in membrane damage repair and autophagy

Early indications for a participation of LGALS3 in membrane integrity surveillance came from its identification as a protein associated to latex bead-containing phagosomes [235]. LGALS3 was also shown to accumulate primarily at the cytosolic face of the limiting membrane of *Mycobacterium*-containing phagosomes [236, 237]. In fact, this lectin gained the status of a protein marker for vacuolar lysis by invasive pathogens [237, 238]. As a member of a group of antibacterial proteins, guanylate-binding protein 2 (GBP2) is attracted by LGALS3 to vacuoles containing pathogens such as *Yersinia* [239]. Called *a sweet way of sensing danger* [240], the physiological relevance of the cytosolic exposure of *N-*glycans upon membrane damage, and their recognition by galectins, become evident when these events were connected with the turnover of the damaged organelle by autophagy [241]. In particular, it was shown that vacuoles ruptured by invading *Salmonella* recruit chimera-type LGALS8, which in turn associates with the autophagy receptor calcium binding and coiled-coil domain 2 (CALCOCO2/NDP52) [241]. Binding between these two proteins is principally mediated by the hydrophobic complementarity of the residues 372–380 of CALCOCO2 and those in the C-terminal CRD of the heterodimeric LGALS8 [242, 243]. This type of pairing is also taking place upon escape of TAU protein aggregates from damaged endosomes, to guarantee cell protection [244]. LGALS8 also interacts with the parkin RBR E3 ubiquitin protein ligase (PRKN), which ubiquitinates the group A *Streptococcus* disrupting the phagosomes and entering the cytoplasm [245]. In contrast, the damage of endocytic compartments caused by Ca^2+^ phosphate precipitates leads to the LGALS3-mediated recruitment of the autophagy receptor SQSTM1 and subsequent engagement of autophagy for lysosomal turnover of the disrupted organelle [246]. The onset of autophagy triggered by endosomal/lysosomal damage also involves tripartite motif containing (TRIM) proteins, especially TRIM16, which associate with LGALS3 that has traced the cytosolic presence of mature *N-*glycans [247]. Since the composition of the cell surface glycome has been shown to determine the extent of galectin binding to disrupted endocytic compartments [248], it makes sense that cells possess more than one galectin to initiate an autophagy response to eliminate damaged endocytic and lysosomal organelles.

**Fig. 5:**
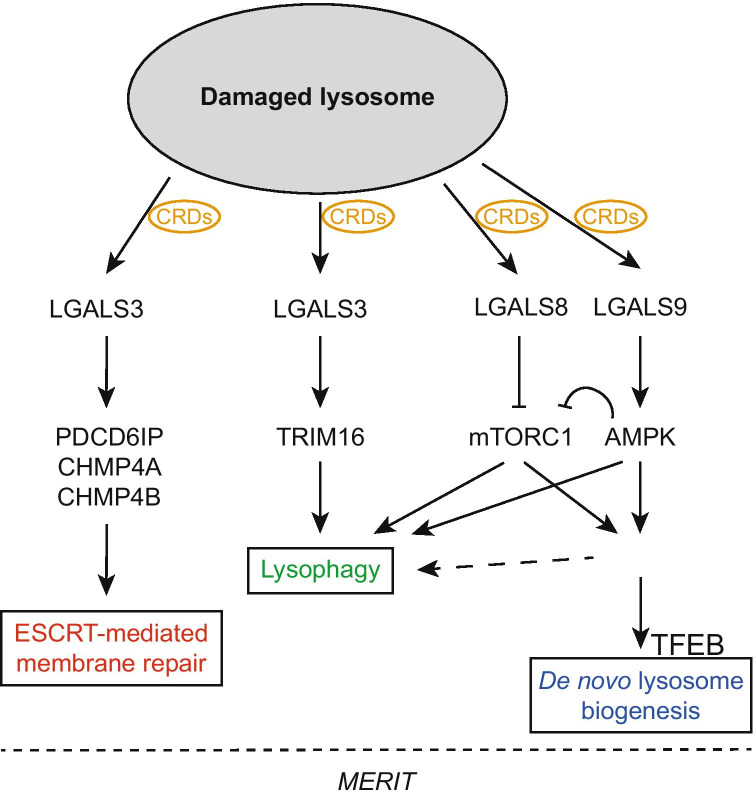
Model illustrating the membrane repair, removal and replacement (MERIT) system. Lysosome membrane damage leads to the cytoplasmic exposure of otherwise not accessible determinants for the cytoplasmic membrane integrity surveillance system. In particular, exposed *N*-glycans are a signal for danger and locally recruit and probably concentrate galectins (LGALS3, 8, 9), which bind the glycans through their carbohydrate recognition domains (CRDs). The bifunctionality of galectins allows them to engage downstream players of the MERIT system, which coordinates lysosomal membrane repair, lysosome removal and replacement. LGALS3 recruits the endosomal sorting complexes required for transport (ESCRT) components programmed cell death 6 interacting protein (PDCD6IP), charged multivesicular body protein 4A (CHMP4A) and CHMP4B, and promotes the formation of ESCRT machinery to repair the damaged lysosomal membranes. LGALS3 also cooperates with tripartite motif containing 16 (TRIM16) to guide autophagy initiation machinery to turnover terminally injured lysosomes. Synergistically, LGALS8 inactivates target of rapamycin complex 1 (mTORC1), while LGALS9 activates AMP-activated kinase (AMPK), which further inhibits mTORC1. The mTORC1 inactivation and AMPK activation lead to cell growth arrest, induction of autophagy and transcription factor EB (TFEB) nuclear translocation to replace damaged lysosomes through de novo biogenesis

The physiological significance of the interaction of cytosolic-exposed mature *N-*glycans and galectins as bifunctional sensors for intracellular membrane integrity is also underscored by their role in the repair and degradation of the compartments of the endolysosomal system upon damage not caused by invading pathogens. LGALS8 is required for the recognition of damaged endosomes by the autophagy (ATG) machinery, so that they are transported by autophagosomes to lysosomes for degradation by a selective type of autophagy known as lysophagy (Fig. 5) [249]. However, LGALS8 and LGALS9 also cooperate to regulate signaling after lysosomal disruption to inhibit mTORC1 and activate AMPK, which triggers both autophagy and the nuclear translocation of TFEB, which both promotes the de novo biogenesis of new lysosomes to replace the degraded ones, and stimulates autophagy (Fig. 5) [250, 251]. Molecularly, LGALS9 displaces the deubiquitinase ubiquitin specific peptidase 9 X-linked (USP9X) from the mitogen-activated protein kinase kinase kinase 7 (MAP3K7/TAK1)-containing complexes, and promotes K63 ubiquitination and activity of MAP3K7, an upstream kinase and AMPK activator [251].

Importantly, a functional connection between galectin and the membrane repair machinery was discerned with the identification of the interaction between LGALS3 and programmed cell death 6 interacting protein (PDCD6IP/ALIX), charged multivesicular body protein 4A (CHMP4A) and CHMP4B, which are components of the ESCRT system (Fig. 5) [252]. The docking of LGALS3 onto the damaged lysosome through binding to the exposed *N-*glycans, leads to the recruitment of PDCD6IP and promotes its interaction with the charged multivesicular body protein 4C (CHMP4C) [253], a downstream effector of the ESCRT-III subcomplex that has the ability to seal damaged membranes [254]. Structurally, the *N*-terminal tail of LGALS3, a section relevant for the lectin’s self-association [255, 256], interacts with the proline-rich region of PDCD6IP [257].

Thus, galectins, due to their dual specificity for glycans and protein effectors, appear to be sensor elements and nucleation core for a system for *lysosomal membrane repair, removal and replacement* (MERIT) (Fig. 5) [258]. In particular, LGALS3 detects membrane damage by binding the cognate part of exposed lumenal *N-*glycans, recruits and organizes ESCRT components at damaged sites on the lysosomes, and facilitates ESCRT-driven repair of the lysosomal membrane (Fig. 5) [253]. At later stages, LGALS3 cooperates with TRIM16 to engage the ATG machinery in the removal of excessively damaged lysosomes through lysophagy [253]. In the absence of LGALS3, repair and autophagy are less efficient, whereas TFEB nuclear translocation increases to compensate lysosomal deficiency via *de novo* lysosomal biogenesis. It remains unclear whether LGALS1, LGALS8 and LGALS9 are multitasking like LGALS3, or whether they are equipping the membrane integrity surveillance system with different types of sensors and/or quality control solutions. Interestingly, the recent discovery of the formation of galectin hybrids via their CRDs may add an additional level of complexity to this regulatory system [259]. Future studies will help deciphering this emerging area of the cell biology of galectins.

## Conclusions and perspectives

Glycans appended to lipids and proteins appear in all the compartments of the secretory and endolysosomal systems, especially the luminal side of their limiting membranes. Our knowledge on the functioning of these systems interconnected by vesicular transport pathways and membrane contact sites has greatly increased through the development of sophisticated cell biological and biochemical tools, such as super-resolution microscopy, correlative light-electron microscopy, mass spectrometry-based proteomics and glycoproteomics, and different high-throughput screening methodologies. Although the transport routes to lysosomes have for long been seen as the digestive system of the cell, numerous recent findings have revealed that the endolysosomal compartments have key roles in the regulation of metabolism and immunity, and as signaling platforms that modulate multiple cell and tissue functions. As a result, glycans and their dynamic modifications represent a mechanism to fine-tune some of these functions, as underlined by the known examples in endocytosis and autophagy highlighted in this review. Indeed, turning cryptic signals into actual signals or switching off activity by a deglycosylation step such as the desialylation of ganglioside GD1a to GM1 [260, 261], are being considered efficient regulatory mechanisms for transport and adhesion [262], or cell growth and differentiation, in this case with galectins or sialic acid-binding immunoglobulin-type lectins (siglecs) as readers of the sugar code. Thus, the mechanics of carbohydrate language is being unraveled, and a dictionary for the relationship between the glycan vocabulary and functions is being set up [263]. As recently predicted, “given the tremendous scientific discoveries in the field lying on and beyond the horizon, life for these future glycobiologists will be sweet indeed” [264].
